# Metabolic syndrome and menopause

**DOI:** 10.1186/2251-6581-12-1

**Published:** 2013-01-03

**Authors:** Zahra Jouyandeh, Farnaz Nayebzadeh, Mostafa Qorbani, Mojgan Asadi

**Affiliations:** 1 Endocrine & Metabolism Research Center, Tehran University of Medical Science, Tehran, Iran; 2 Tehran Women General Hospital, Tehran University of Medical Science, Tehran, Iran; 3 Department of Epidemiology & Biostatistics, School of Public Health, Tehran University of Medical Sciences, Tehran, Iran

**Keywords:** Menopause, Metabolic syndrome, Prevalence

## Abstract

**Background:**

The metabolic syndrome is defined as an assemblage of risk factors for cardiovascular diseases, and menopause is associated with an increase in metabolic syndrome prevalence. The aim of this study was to assess the prevalence of metabolic syndrome and its components among postmenopausal women in Tehran, Iran.

**Methods:**

In this cross-sectional study in menopause clinic in Tehran, 118 postmenopausal women were investigated. We used the adult treatment panel 3 (ATP3) criteria to classify subjects as having metabolic syndrome.

**Results:**

Total prevalence of metabolic syndrome among our subjects was 30.1%. Waist circumference, HDL-cholesterol, fasting blood glucose, diastolic blood pressure ,Systolic blood pressure, and triglyceride were significantly higher among women with metabolic syndrome (P-value<0.05). Our study shows high abdominal obesity and hypertension are the most prevalent components of metabolic syndrome. 15%, 13.3% and 1.8% of subjects had three, four and five criteria for metabolic syndrome, respectively. There was a significant relationship between number of components of metabolic syndrome and waist circumference.

**Conclusions:**

Our study shows that postmenopausal status is associated with an increased risk of metabolic syndrome. Therefore, to prevent cardiovascular disease there is a need to evaluate metabolic syndrome and its components from the time of the menopause.

## Background

Metabolic syndrome is an assemblage of several factors including hypertension, dyslipidemia, insulin resistance, obesity and glucose intolerance that increase subjects' risk to develop cardiovascular disease (CVD) and type 2 diabetes [1, 2]. Diagnostic criteria for metabolic syndrome has been defined by the national cholesterol education program adult treatment panel ΙΠ (ATP ΙΠ), which is easily used for classifying patients [3]. It's estimated that almost 20-30% of the middle-aged population are affected by this syndrome [4] varies from 8 to 24% in males [5, 6] and from 7 to 46% in females [7, 8]. Many cross-sectional studies have shown an increased risk of metabolic syndrome in postmenopausal women which varies from 32.6% to 41.5% [9–11]. Some studies show an increasing prevalence of metabolic syndrome in developing countries and Asia [12] and an estimated prevalence in Iran from 35-58% [13, 14]. As there is not enough data on the prevalence of metabolic syndrome among postmenopausal women in Tehran referral hospitals, we decided to establish a study in menopause clinic of Tehran women general hospital to determine the prevalence of metabolic syndrome and its components in postmenopausal women.

## Methods

This cross-sectional study was performed in menopause clinic of Tehran women general hospital. The study was performed on 118 postmenopausal women from January 2011 to January 2012 in the menopause clinic. Menopause was defined as at least 12 consecutive months of amenorrhea with no other medical cause. Exclusion criteria were considered as surgical menopause and chemo radiotherapy. No other conditions or disease have been considered. The women mostly were visited in the clinic because of hot flashes, mood swing, vaginal dryness, sleep disturbances, night sweat, forgetfulness, urinary symptoms, pain with intercourse, palpitations, anxiety, joint and muscle pain, depression and irritability.

A questionnaire was completed for each patient including demographic information, menopausal status, medical history, reproductive history, drug history, family history, physical examination and clinical lab data. An inform consent was signed by each patient in the clinic. Postmenopausal women were considered to have metabolic syndrome if they had any three or more of the following criteria, according to the ATP ΙΠ criteria [2]:

1. Abdominal obesity: waist circumference≥ 88 cm
2. Hypertriglyceridemia: serum TG level ≥ 150 mg/dl
3. Serum HDL: < 50 mg/dl
4. High blood pressure: SBP ≥ 130 mmHg and/or DBP ≥ 85 mmHg or on treatment for hypertension
5. High fasting glucose: serum glucose level > 110 mg/dl or on treatment for diabetes

Waist circumference was measured at a level midpoint between the lower rib margin and the top of the iliac crest. Blood pressure of the patients was measured twice with a standard barometer in a sitting position, and the average blood pressure had been documented in the sheets. All data were analyzed by SPSS version 16 (SPSS Inc, Chicago, IL, USA). The continuous variables are reported as Mean ± SD and categorical variables are presented as percentage. The distribution of continuous variables was assessed by Kolomogrov-Smirnov test and it demonstrated a normal distribution. A P-value<0.05 was considered statistically significant.

## Results

A total of 118 postmenopausal women were studied. Table 1 shows the baseline characteristics of these women with and without metabolic syndrome. The mean age of our subjects was 52.67 ± 5 years and the mean age of menopause was 47.66 ± 4.44 years. Total prevalence of metabolic syndrome among our subjects was 30.1%.

**Table 1:** **Baseline data of postmenopausal women with and without metabolic syndrome**

P- value	Subjects without metabolic syndrome	Subjects with metabolic syndrome	Total number of subjects	Parameters
0.272	52.56±4.81	53.61±4.51	52.67±5	Age(years)
0.315	47.62±4.31	48.47±3.93	47.66±4.44	Menopausal age(years)
0.001	88.34±9.20	94.91±8.89	90.45±9.56	WC(cm)
0.042	118.79±18.78	125.91±15.83	120.64±18.08	SBP(mmHg)
0.003	79.81±11.33	85±7.48	81.06±10.54	DBP(mmHg)
0.001	99.82±30.86	111.20±29.20	103.35±30.35	FBS(mg/dl)
<0.05	124.68±53.50	200.64±103.99	146.92±79.94	TG(mg/dl)
0.571	215.06±39.97	211.08±31.12	213.83±37.16	Total Cholesterol(mg/dl)
<0.05	58.20±12.11	47.35±8.22	55.03±12.04	HDL(mg/dl)
0.408	128.94±31.24	124.20±26.14	127.36±29.37	LDL(mg/dl)

*WC: waist circumference, SBP: systolic blood pressure, DBP:diastolic blood pressure, FBS:fasting blood glucose, TG: triglyceride, HDL:high density lipoprotein, LDL: low density lipoprotein.

**P-value<0.05 was considered statistically significant.

Waist circumference, HDL-cholesterol, fasting blood glucose, diastolic blood pressure ,Systolic blood pressure, and triglyceride were significantly higher among women with metabolic syndrome (P-value<0.05). There were no significant differences in the age, menopausal age, total cholesterol and LDL-cholesterol. The percentage of fasting blood sugar>110 mg/dl, high density lipoprotein<50 mg/dl, Triglyceride≥150 mg/dl, waist circumference≥88 cm, and systolic blood pressure ≥130 mmHg/diastolic blood pre-ssure≥85 mmHg were 29.1%, 35.6%, 35.6%, 64.3%, 47.9% respectively. The percentages of each metabolic syndrome components are shown in Table 2. Table 3 shows prevalence of subjects with criteria of metabolic syndrome which shows 15%, 13.3% and 1.8% had three, four and five criteria for metabolic syndrome respectively. There was a significant relevancy between number of metabolic syndrome components and increasing waist circumference (P-value: 0.001) with a statistically significant linearity (<0.05).

**Table 2:** **Prevalence of metabolic syndrome and the components of metabolic syndrome in postmenopausal women**

30.1%	Metabolic syndrome
64.3%	Waist circumference≥88 cm
35.6%	Triglyceride≥150 mg/dl
35.6%	HDL-cholesterol<50 mg/dl
47.9%	Systolic blood pressure≥130 mmHg/diastolic blood pressure≥85 mmHg
29.1%	Fasting blood glucose>110 mg/dl

**Table 3:** **Number of metabolic syndrome components versus waist circumference**

P-value	Five	Four	Three	Two	One	None	Metabolic syndrome components	
0.001	105.50±2.12	94.26±6.80	94.23±10.37	89.94±9.29	89.03±10.01	82.64±4.32	Mean±SD	Waist circumference
	1.8	13.3	15	33.6	23.9	12.4	Percent	

## Discussion

In our study the overall prevalence of metabolic syndrome was 30.1% among postmenopausal women. Other studies report a prevalence of metabolic syndrome near to our results. A cross-sectional study in Gorgan province in Iran shows a prevalence of 31% [15] which was similar to our findings. Other Studies in Austria, China, Germany, Iran and Canada showed a prevalence of 32.6%, 37.34%, 36.1%, 31% and 29.6% respectively [9, 16–19] in an agreement with our finding. Although there was a disagreement between our study and some other studies done in Iran, western India, Argentina and Ecuador with a prevalence of 69%, 55%, 22% and 41.5% respectively [20–23]. These differences in prevalence of metabolic syndrome in different studies can be due to different investigation methods of the syndrome (different investigation criteria), socioeconomic and environmental differences, genetic factors and lifestyle. In our study, we found that waist circumference, systolic blood pressure, diastolic blood pressure, fasting blood sugar, triglyceride and HDL levels were significantly higher among postmenopausal women with metabolic syndrome in comparison to postmenopausal women without metabolic syndrome. The most prevalent component of metabolic syndrome was abdominal obesity with a frequency of 64.3%, which is in agreement with studies in north east of Iran [24], Babol [18] and Argentina [25] about the most prevalent component of metabolic syndrome among postmenopausal women with metabolic syndrome. This is not exactly similar to the findings of other studies in Ecuador [23] with high TG level and in Korea [26], Brazil [25] and Iran [15] with low HDL-cholesterol level reported as the most prevalent component of metabolic syndrome. These differences may be due to genetic, ethnic and lifestyle differences in these countries. Table 4 shows the prevalence of metabolic syndrome and its components done in different studies over the world.

**Table 4:** **Prevalence of metabolic syndrome and its components in different studies**[15, 18, 22–27]

Study		Number	Metabolic syndrome	Htn	High TG	Impaired FBS	Low HDL	High WC
Iran	Gorgan	100	31%	16%	16%	17%	30%	29%
	North East	160	20.62%	3.12%	20%	14.30%	29.37%	34.37%
	Babol	984	31%	12.1%	41.5%	12.1%	48.6%	76.6%
	Tehran	940	53.5%	16.5%	104.4%	66.2%	12.5%	9.8%
Brazil		323	44.4%	65%	12.4%	11.8%	79.6%	50.9%
Argentina		124	22%	8%	8%	2%	10%	13%
Ecuador		325	41.5%	65.9%	83%	29.6%	80%	83.7%
Korea		778	54.6%	63.6%	39.3%	26%	69.8%	67.1%

We found a statistically significant relationship between waist circumference and number of metabolic syndrome components, which was in agreement with study done by Marjani et al. in Gorgan [15]. Abdominal obesity is a risk factor for cardiovascular disorders [28, 29] and can cause metabolism abnormality and threaten human’s health [30]. Therefore, it is necessary to reduce this risk among postmenopausal women with metabolic syndrome by changing the lifestyle leading to weight loss by a healthy diet and frequent physical activity.

In our study we found that both systolic and diastolic blood pressure was higher among post menopausal women with metabolic syndrome specially DBP. Marjani et al. also showed a significantly high diastolic blood pressure among postmenopausal women in Gorgan [15].This may suggest us that diastolic blood pressure is a risk factor for CHD in postmenopausal women with metabolic syndrome that should be considered.

A significant difference of impaired fasting glucose was found among postmenopausal women with and without metabolic syndrome in our study. Walton and colleges also report an increase in FBS among postmenopausal women with metabolic syndrome [31].

Finally, Our findings show low HDL and high TG levels in postmenopausal women with metabolic syndrome, which is in agreement with findings in studies done by Marjani et al. [15] in Iran and Figueiredo Neto et al. [25] in Brazil. There are controversial findings about menopausal effect on HDL [32, 33] and TG levels [32, 34]. Our findings about high prevalence of dyslipidemia among postmenopausal women with metabolic syndrome indicate a need to treat metabolic syndrome in postmenopausal women as a target for reducing cardiovascular risks with an special effort on lifestyle changing and daily diets.

A limitation of this study was the small population studied, done as a cross-sectional study which may limit generalization of this study to all parts of Iran. There is a need of further studies to confirm the results found and, then, take actions to prevent metabolic syndrome in postmenopausal women.

## Conclusion

Our study has shown a high prevalence of metabolic syndrome among postmenopausal women referring to menopause clinic in Tehran women general hospital that abdominal obesity and hypertension were the most prevalent components of metabolic syndrome among these patients. These components can lead to an increase in cardiovascular diseases. Interventions are needed to modify these risk factors such as abdominal obesity, dyslipidemia, hypertension and glucose intolerance and reduce the risk of cardiovascular events. Therefore, it is important to have more efforts for lipid screening and educational programs to improve women’s knowledge about a healthy lifestyle.

## References

[1] Miranda PJ, DeFronzo RA, Califf RM, Guyton JR (2005). Metabolic syndrome: definition, pathophysiology, and mechanisms. Am Heart J.

[2] Expert Panel on DetectionEvaluation THBCA (2001). Executive summary of the third report of the National Cholesterol Education Program (NCEP) expert panel on detection, evaluation, and treatment of high blood cholesterol in adults (adult treatment panel III). J-AM MED ASSOC.

[3] Grundy SM, Cleeman JI, Daniels SR, Donato KA, Eckel RH, Franklin BA (2005). Diagnosis and management of the metabolic syndrome. Circulation.

[4] Meigs JB (2002). Epidemiology of the metabolic syndrome, 2002. Am J Managed Care.

[5] Gupta A, Gupta R, Sarna M, Rastogi S, Gupta V, Kothari K (2003). Prevalence of diabetes, impaired fasting glucose and insulin resistance syndrome in an urban Indian population. Diabetes Res Clin Pract.

[6] Ford ES, Giles WH, Dietz WH (2002). Prevalence of the metabolic syndrome among US adults. JAMA: J Am Med Assoc.

[7] Balkau B, Vernay M, Mhamdi L, Novak M, Arondel D, Vol S (2003). The incidence and persistence of the NCEP (National Cholesterol Education Program) metabolic syndrome. The French DESIR study. Diabetes & Metabolism.

[8] Ramachandran A, Snehalatha C, Satyavani K, Sivasankari S, Vijay V (2003). Metabolic syndrome in urban Asian Indian adults—a population study using modified ATP III criteria. Diabetes Res Clin Pract.

[9] Ponholzer A, Temml C, Rauchenwald M, Marszalek M, Madersbacher S (2007). Is the metabolic syndrome a risk factor for female sexual dysfunction in sexually active women?. Int J Impotence Res.

[10] Chedraui P, Hidalgo L, Chavez D, Morocho N, Alvarado M, Huc A (2007). Quality of life among postmenopausal Ecuadorian women participating in a metabolic syndrome screening program. Maturitas.

[11] Park YW, Zhu S, Palaniappan L, Heshka S, Carnethon MR, Heymsfield SB (2003). The metabolic syndrome: prevalence and associated risk factor findings in the US population from the Third National Health and Nutrition Examination Survey, 1988–1994. Archives Internal Med.

[12] Meigs JB (2000). Invited commentary: insulin resistance syndrome? Syndrome X? Multiple metabolic syndrome? A syndrome at all? Factor analysis reveals patterns in the fabric of correlated metabolic risk factors. Am J Epidemiol.

[13] Azizi F, Salehi P, Etemadi A, Zahedi-Asl S (2003). Prevalence of metabolic syndrome in an urban population: Tehran Lipid and Glucose Study. Diabetes Res Clin Pract.

[14] Sarrafzadegan N, Kelishadi R, Baghaei A, Hussein Sadri G, Malekafzali H, Mohammadifard N (2008). Metabolic syndrome: an emerging public health problem in Iranian women: Isfahan Healthy Heart Program. Int J Cardiology.

[15] Marjani A, Moghasemi S (2012). The Metabolic Syndrome among Postmenopausal Women in Gorgan. Int J Endocrinol.

[16] Ding QF, Hayashi T, Zhang XJ, Funami J, Ge L, Li J (2007). Risks of CHD identified by different criteria of metabolic syndrome and related changes of adipocytokines in elderly postmenopausal women. J Diabetes Complications.

[17] Deibert P, König D, Vitolins MZ, Landmann U, Frey I, Zahradnik HP (2007). Effect of a weight loss intervention on anthropometric measures and metabolic risk factors in pre-versus postmenopausal women. Nutr J.

[18] Delavar MA, Lye MS, Khor GL, Hanachi P, Syed Hassan ST, Delavar MA, Lye MS, Khor GL, Hanachi P, Syed Hassan STB (2009). Prevalence of metabolic syndrome among middle aged women in Babol, Iran. Southeast Asian J Tropical Med Public Health.

[19] Piché MÈ, Weisnagel SJ, Corneau L, Nadeau A, Bergeron J, Lemieux S (2006). The WHO and NCEP/ATPIII definitions of the metabolic syndrome in postmenopausal women: are they so different?. Metabolic Syndrome Related Disord.

[20] Ainy E, Mirmiran P, Zahedi Asl S, Azizi F (2007). Prevalence of metabolic syndrome during menopausal transition Tehranian women: Tehran Lipid and Glucose Study (TLGS). Maturitas.

[21] Pandey S, Srinivas M, Agashe S, Joshi J, Galvankar P, Prakasam C (2010). Menopause and metabolic syndrome: a study of 498 urban women from western India. J Mid-life Health.

[22] Mesch V, Boero L, Siseles N, Royer M, Prada M, Sayegh F (2006). Metabolic syndrome throughout the menopausal transition: influence of age and menopausal status. Climacteric.

[23] Hidalgo LA, Chedraui PA, Morocho N, Alvarado M, Chavez D, Huc A (2006). The metabolic syndrome among postmenopausal women in Ecuador. Gynecological Endocrinol.

[24] Marjani A, Hezarkhani S, Shahini N (2012). Prevalence of Metabolic Syndrome among Fars Ethnic Women in North East of Iran. World J Med Sci.

[25] Figueiredo Neto JA, Figuerêdo ED, Barbosa JB, Barbosa FF, Costa GRC, Nina VJS (2010). Metabolic syndrome and menopause: Cross-sectional study in gynecology clinic. Arq Bras Cardiol.

[26] Kim HM, Park J, Ryu SY, Kim J (2007). The effect of menopause on the metabolic syndrome among Korean women. Diabetes Care.

[27] Eshtiaghi R, Esteghamati A, Nakhjavani M (2010). Menopause is an independent predictor of metabolic syndrome in Iranian women. Maturitas.

[28] Kannel WB, Adrienne Cupples L, Ramaswami R, Stokes J, Kreger BE, Higgins M (1991). Regional obesity and risk of cardiovascular disease; the Framingham Study. J Clin Epidemiol.

[29] Rexrode KM, Carey VJ, Hennekens CH, Walters EE, Colditz GA, Stampfer MJ (1998). Abdominal adiposity and coronary heart disease in women. JAMA: J Am Med Assoc.

[30] Lobo RA (2008). Metabolic syndrome after menopause and the role of hormones. Maturitas.

[31] Walton C, Godsland I, Proudler A, Wynn V, Stevenson J (1993). The effects of the menopause on insulin sensitivity, secretion and elimination in non‒obese, healthy women. European J Clin Invest.

[32] Jensen J, Nilas L, Christiansen C (1990). Influence of menopause on serum lipids and lipoproteins. Maturitas.

[33] Do K, Green A, Guthrie J, Dudley E, Burger H, Dennerstein L (2000). Longitudinal study of risk factors for coronary heart disease across the menopausal transition. Am J Epidemiol.

[34] Peters H, Westendorp I, Hak A, Grobbee D, Stehouwer C, Hofman A (1999). Menopausal status and risk factors for cardiovascular disease. J Internal Med.

